# Rapid activation of hematopoietic stem cells

**DOI:** 10.1186/s13287-023-03377-6

**Published:** 2023-06-06

**Authors:** Roshina Thapa, Erez Elfassy, Leonid Olender, Omri Sharabi, Roi Gazit

**Affiliations:** The Shraga Segal Department of Microbiology, Immunology, and Genetics, Faculty of Health Sciences, National Institute for Biotechnology in the Negev, The Ben-Gurion University of the Negev, 84105 Beer Sheva, Israel

**Keywords:** Hematopoietic stem cells, HSC, Activation markers, Rapid activation, CD69, CD317, BST2, Ki67, Differentiation, Proliferation

## Abstract

Adult hematopoietic stem cells (HSCs) in the bone marrow (BM) are quiescent. Following perturbations, such as blood loss or infection, HSCs may undergo activation. Surprisingly, little is known about the earliest stages of HSCs activation. We utilize surface markers of HSCs activation, CD69 and CD317, revealing a response as early as 2 h after stimulation. The dynamic expression of HSCs activation markers varies between viral-like (poly-Inosinic-poly-Cytidylic) or bacterial-like (Lipopolysaccharide) immune stimuli. We further quantify dose response, revealing a low threshold, and similar sensitivity of HSCs and progenitors in the BM. Finally, we find a positive correlation between the expression of surface activation markers and early exit from quiescence. Our data show that the response of adult stem cells to immune stimulation is rapid and sensitive, rapidly leading HSCs out of quiescence.

**Supplementary Information:**

The online version contains supplementary material available at 10.1186/s13287-023-03377-6.

## Introduction

Hematopoietic stem cells (HSCs) are the source of blood and immune cells [1, 2]. HSCs are largely quiescent in the adult bone marrow (BM), preserving long-term multipotency [3–6]. When needed, HSCs may undergo activation and presumably contribute to accelerated hematopoiesis [7–10]. The speed and the sensitivity threshold of HSCs activation are yet to be determined.

Poly-Inosinic-poly-Cytidylic (pIpC) and Lipopolysaccharide (LPS) are the most commonly used viral- and bacterial-like immune stimuli, respectively [11]. pIpC induces type-I interferon response, activating HSCs in the BM [12, 13]. Such HSCs activation depends largely on IFNα [12, 14]. Interestingly, pIpC may also skew HSCs differentiation into megakaryocytes [15], presumably to enhance coagulation. Moreover, chronic pIpC stimulation drives DNA damage and exhaustion of genetically susceptible HSCs [16], while chronic viral infection impairs the potency of HSCs indirectly via CD8 T cells that change the BM niche [17]. LPS stimulates the major TLR4 pathway, and activates HSCs directly and indirectly via major inflammatory cytokines such as IL-1β, IL-6, TNF-α, and IFN-γ [8, 18].

Overwhelming stimulation can exhaust HSCs [19–22]; however, following milder simulations HSCs re-enter quiescence and preserve potency, even with prolonged stimulation [23]. Interestingly, LPS and related cytokines can bias differentiation into the myeloid rather than the lymphoid lineage [18, 22, 24]. Immune stimulation was further reported to elicit trained immunity in HSCs, including metabolic and epigenetic myeloid skewing [25, 26]. Both viral- and bacterial stimuli induce the activation of HSCs – and may give rise to more effector cells [8, 14, 16, 18, 23, 27]. Surprisingly, previous studies focused primarily on phenotypes emerging within days and months, while earlier stages of HSCs activation have not been studied extensively.

We previously identified HSC-activation markers [28], including CD69 (*Clec2c*) and CD317 (*Bst2*). These markers were validated in a study showing the essential role of CD317 in retaining activated HSCs in the BM niche [29]. In the current work, we sought to utilize these surface markers to study how early is the activation of HSCs, and how sensitive are HSCs to commonly used immune stimuli. Our quantified data show rapid response within 2 h, and high sensitivity down to 0.1 µg, with a positive correlation between activation markers and the exit from dormancy of HSCs in the BM.

## Materials and methods

### Mice and ethical

All mice were kept at the Ben-Gurion University SPF (specific pathogen-free) unit. Mice strains used were C57Bl/6 J. Both male and female mice at 8–12 weeks old were used for the experiments, with age- and gender-matched littermates as controls. All experiments were carried out according to the guidelines of the ethical committee and after approval of the local and state IACUC (IL-68-11-2018-D).

### Immune stimulation

Mice were stimulated by poly-Inosinic–poly-Cytidylic acid sodium (pIpC: Invivogen, High Molecular Weight, catalog code: trl-pic-5) intraperitoneally (i.p.) 100 µg per mouse. Lipopolysaccharides of E. coli O55:B5 (LPS, Sigma-Aldrich cat: L2880 lot: 113M4068V) were administered i.p. at 20 µg per mouse. Stimulation was done at various time points (2, 4, and 24 h). Unstimulated mice were used as control, such as time 0. For dose response, mice were stimulated by pIpC with doses of 0.01, 0.1, 1, and 10 µg, then analyzed at 24 h. LPS administered i.p. with doses of 0.0001, 0.001, 0.01, 0.1, and 1 µg per mouse, then analyzed at 2 h. Unstimulated mice were used as control.

### Isolation of cells and flow cytometric analysis

Mice were euthanized by CO_2_ or terminal isoflurane. Bone marrow cells were extracted from the tibia, femur, and pelvis using mortar and pestle; sample media comprised of Phosphate Buffer Saline (PBS) with 2 mM EDTA and 2% Foetal Bovine Serum. Mononuclear cells were enriched over Ficoll-Paque™ 1.084 (Cytiva, cat #17544602). All antibodies are from Biolegend: Lineage Cocktail- PacBlue Cat# 133310, Lineage-A700 Cat# 652419, Sca1-APC Cat# 108112, cKit-APCcy7 Cat# 105826 CD150-PeCy7 Cat#115914, CD48-PerCP/cyanine5.5 Cat# 103422, CD69-FITC Cat# 104506, CD317-PE Cat# 127010, DAPI Cat# 422801; Ki67-FITC Cat# 652410, Ki67-PE Cat# 652404. Cytoflex LX (Beckman Coulter) was used to acquire data. Cytexpert software was used to visualize and analyze FACS data.

### Cell cycle analysis

BM cells were enriched as above and stained: Lineage-A700, Sca1-APC, cKit-APCCy7, CD150-PECy7, CD48- PC5.5, CD69-FITC, and CD317-PE. Cells were washed, fixated in a 96U plate in 250 µl PBS × 1 at room temperature with 2% paraformaldehyde (PFA, EMS #15710) for 20 min, washed twice with PBS × 1, permeabilized in 0.25%Tween 20 (Sigma-Aldrich, P1379) in PBS × 1 for 30 min, washed twice in 0.1% tween PBS × 1, then stained by Ki67-FITC or Ki67-PE in 1:300 of stock in 0.1% tween PBS overnight. DAPI (10 µg/ml) was added to the cells before flow-cytometric analysis.

### Statistics

FACS data are shown as mean ± SD. Data are representative of at least three independent experiments unless otherwise noted. A two-tailed T-test was performed, with *p* < 0.05 considered significant. Linear regression statistical tool was performed using the Graphpad prism v5.

## Results

### Immune activation of hematopoietic stem cells is rapid

**Fig. 1:**
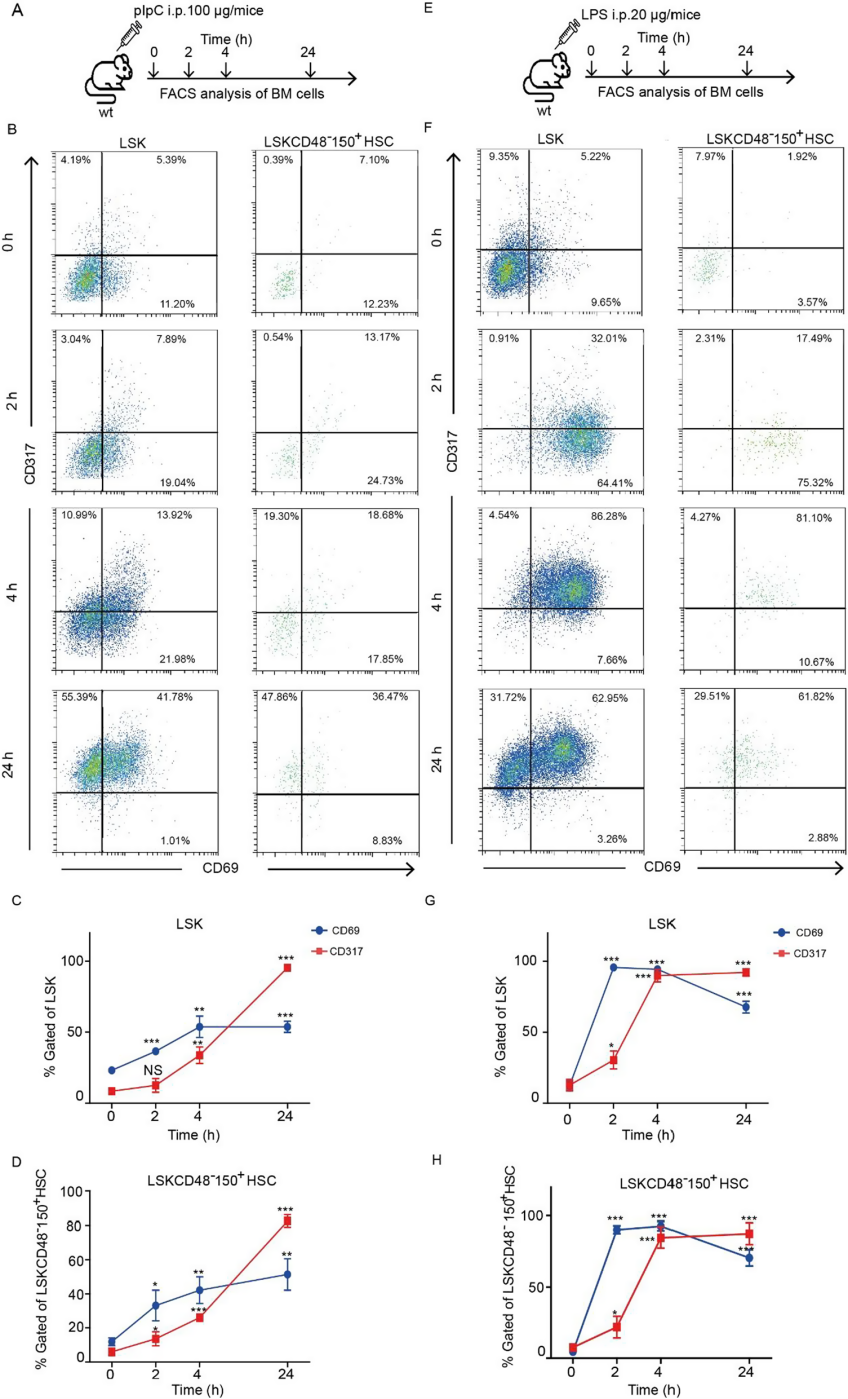
Immune activation of hematopoietic stem and progenitor cells is rapid. **A** Schematic representation of the experimental settings involving injection of pIpC at different time points (0, 2, 4, and 24 h). **B** Representative FACS plots showing the percentage of CD69^+^ and CD317^+^ cells in the LSK (left panels) and LSKCD48^−^150^+^ HSCs (right panels) populations from control and pIpC stimulated mice at different time points. **C**, **D** Quantification of CD69 and CD317 expression in the LSK and LSKCD48^−^150^+^ HSCs populations in the BM of control and pIpC stimulated mice. **E** Schematic representation of the experimental settings involving injection of LPS at different time points (0, 2, 4, and 24 h). **F** Representative FACS plots showing the percentage of CD69^+^ and CD317^+^ cells in the LSK (left panels) and LSKCD48^−^150^+^ HSCs (right panels) populations from control and LPS-stimulated mice at different time points. **G**, **H** Quantification of CD69 and CD317 expression in the LSK and LSKCD48^−^150^+^ HSCs populations in the BM of control and LPS-stimulated mice. *p < 0.05, **p < 0.01, ***p < 0.001

We previously showed that HSCs activation markers CD69 and CD317 are brightly expressed 24 h following immune stimulation [28]. However, we did not find data on HSCs response at earlier time points. Refining the time scale analysis of HSCs activation may reveal how fast is their response. Therefore, we stimulated mice with pIpC and collected BM at 2, 4, and 24 h (Fig. 1A). We observed an elevation of CD69 expression to more than 30% of the Lineage^−^Sca1^+^cKit^+^ compartment (LSK, encompassing multipotent stem- and progenitors), and in the LSKCD48^−^CD150^+^ HSCs population after 2 h (Fig. 1B). CD69 expression further increased at 4 and 24 h (Fig. 1C, D). Intriguingly, CD317 expression increased slower than CD69 at 2 and 4 h, with a peak at 24 h (Fig. 1C, D). Progenitors showed a similar trend; increased CD317 expression on Lineage-negative (Lin^−^), or Lin^−^cKit^+^ (LK) populations occurred at a comparable time-dependent scale (Additional file 1: Fig. S1). Notably, the LSK and LSKCD48^−^150^+^ HSCs showed overall higher frequencies of CD69 and CD317, but with a similar dynamic. In the case of pIpC stimulation, after 24 h, more than 80% of LSKCD48^−^CD150^+^ HSCs, and almost 100% of LSK cells elevated their CD317 expression. On the other hand, less than 60% of Lin^−^ and less than 80% of LK cells expressed CD317 at the same time point (Additional file 1: Fig. S1 A–C). Importantly, the pIpC stimulation also induced elevation of Sca1, shifting more progenitors into the LSK gate at 24 h after stimulation, but not as much at 2 or 4 h (data not shown). We also wanted to examine a bacterial-type stimulation. We injected intra-peritoneal 20 µg LPS (Fig. 1E). CD69 expression was robust already at 2 h in both the LSK and the LSKCD48^−^CD150^+^ HSCs populations (Fig. 1G). Interestingly, following LPS stimulation, CD317 reached similar expression levels only at 4 h. Later, CD69 decreased, whereas CD317 levels were maintained in most of the cells (Fig. 1F–H). Furthermore, the elevation of CD69 and CD317 in other populations in the BM of the same mice was also substantial (Additional file 1: Fig. S1 D–F). Lin^−^ population followed the same time-dependent trend in expression of both CD69 and CD317, peaking at 2 and 4 h after stimulation and reaching about 40% of total cells. This peak was followed by a decrease in surface levels of CD69, in agreement with previously published data [28]. In the case of the LK population, the baseline of CD69 expression was already at about 25% of the cells, with a peak of about 60% at 4 h. CD317 elevated after 4 h to about 90% of the cells. The expression of both CD69 and CD317 decreased after 24 h, with CD69 levels returning almost to baseline levels and CD317 remaining at about 60% (Additional file 1: Fig. S1 D–F). Notably, LPS stimulation, like pIpC stimulation, caused an elevation of Sca1 expression, suggesting that some of the cells with the LSK immune phenotype may have increased expression as a direct result of the immune stimulation. Our data show CD69 to be more responsive during the earlier phase, whereas CD317 elevate later. Surprisingly, the frequencies of CD69 and CD317 in HSCs were higher than in other progenitors populations.

### CD317 reveals a dose–response to pIpC above a minimal threshold

**Fig. 2:**
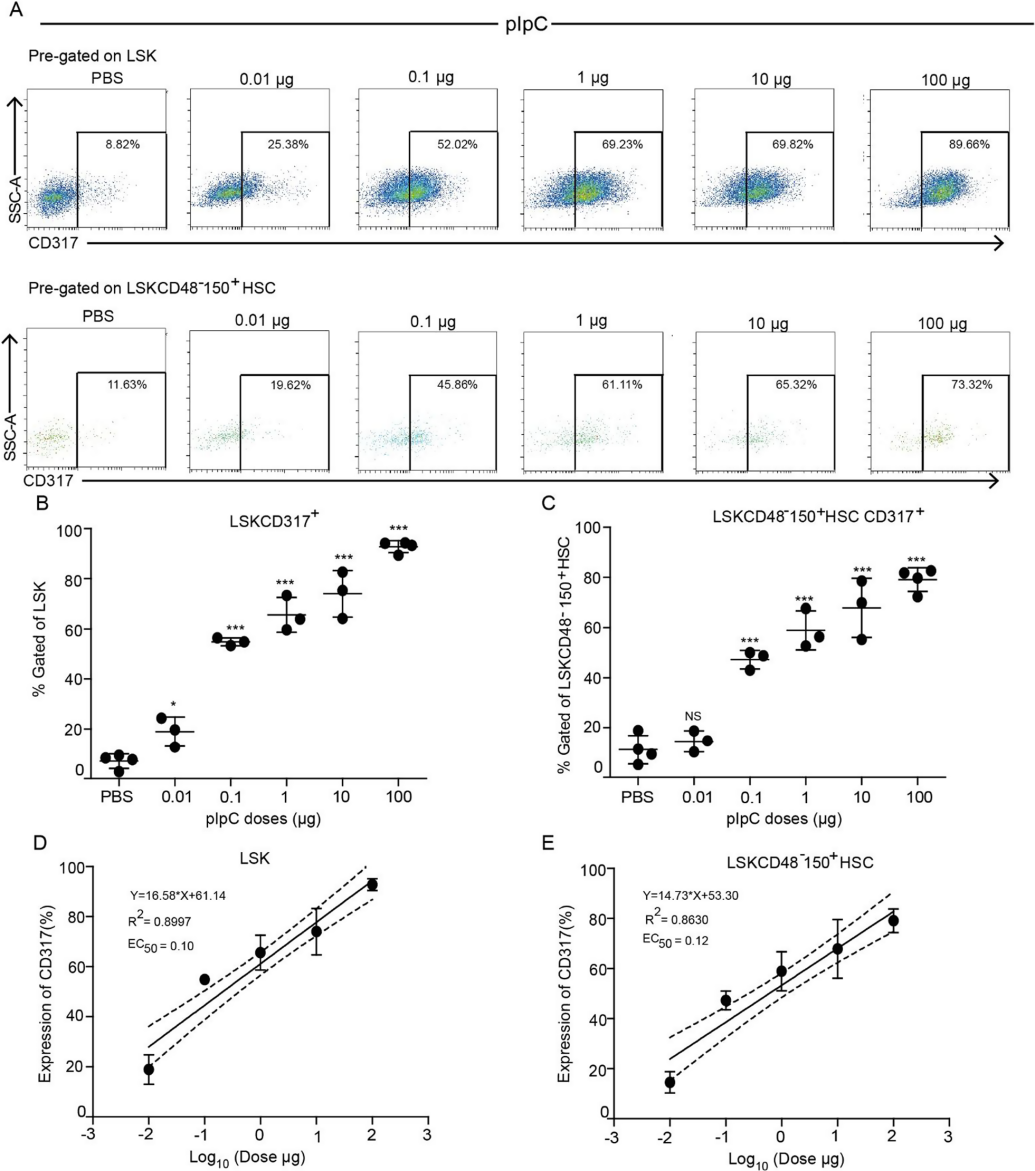
CD317 reveals a dose–response to pIpC above a minimal threshold. **A** Representative FACS plots showing the percentage of CD317^+^ cells in the LSK (upper panels) and LSKCD48^−^150^+^ HSCs (lower panels) populations from the BM of PBS-treated (control) and pIpC- stimulated mice under various pIpC doses (0.01–100 µg as positive control) at 24 h post-injection. **B**, **C** Quantification of the LSK CD317^+^ and LSKCD48^−^150^+^ HSCs CD317^+^ cell populations from the BM of PBS-treated (control) mice and mice treated with different doses of pIpC. **D**, **E** Linear regression model fitting the dose-dependent response with the expression of CD317 in LSK and LSKCD48^−^150^+^ HSCs populations. Solid lines represent the linear fit of data. Dotted lines represent 95% confidence intervals. Data are presented as average ± SD, a summary of three independent experiments, *n* ≥ 3 mice per group; *p < 0.05, **p < 0.01, ***p < 0.001

Following the findings of HSCs rapid response to pIpC and accumulation of CD317 at 24 h, we next wanted to examine if HSCs activation is dose-dependent or rather an all-or-none response. To test this hypothesis, we injected mice with various doses of pIpC, ranging from 0.01 to 100 µg, and analyzed the expression of CD317 in different populations of BM hematopoietic cells. The CD317 expression in the LSK and the LSKCD48^−^CD150^+^ HSCs was proportional to an increasing dose of pIpC, indicating 0.1 µg of pIpC as an effective dose for substantial induction of CD317 expression. An increase in the dose of pIpC led to an increment in the expression of CD317 (Fig. 2A–C; Additional file 1: Fig S2 F). The pIpC dose was further confirmed by the EC50 calculation, showing a good fit with a linear model: 0.1 µg for LSK and 0.12 µg for LSKCD48^−^CD150^+^ HSCs (Fig. 2D, E). Similarly, Lin^−^ or LK cells followed a dose–response trend for CD317 expression with pIpC (Additional file 1: Fig. S2 A–C). However, the maximal increment observed within the Lin^−^ population reached only 40%, and LK only 60%, while LSK cells and the LSKCD48^−^CD150^+^ HSCs gained > 80% positive cells, and higher MFI values (Additional file 1: Fig. S2 A–C, F). Furthermore, calculations of the EC50 values for the CD317 activation in Lin^−^ and LK populations fit well with a linear expression model and were found to be 0.07 µg and 1.30 µg, respectively (Additional file 1: Fig. S2 D, E). Therefore, hematopoietic stem- and progenitor cells have a dose-dependent expression of surface CD317 in response to a broad range of pIpC concentrations.

### CD69 elevation is dose-dependent at higher concentrations of LPS stimulation

**Fig. 3:**
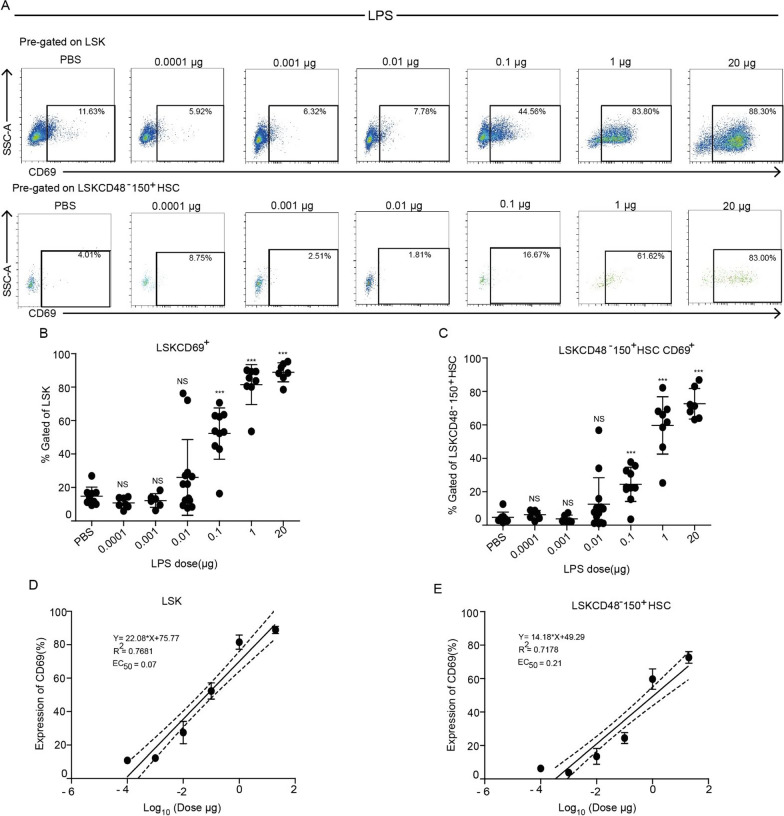
CD69 elevation is dose-dependent at higher concentrations of LPS. **A** Representative FACS plots showing the percentage of CD69^+^ cells in the LSK (upper panels) and LSKCD48^−^150^+^ HSCs (lower panels) populations from the BM of PBS-treated (control) mice and mice stimulated with various doses of LPS (0.0001–20 µg) at 2 h post-injection. **B**, **C** Quantification of the LSK CD69^+^ and LSKCD48^−^150^+^ HSCs CD69^+^ cell populations from the BM of PBS-treated (control) mice and mice treated with different doses of LPS. **D**, **E** Linear regression model fitting the dose-dependent response with the expression of CD69 in LSK and LSKCD48^−^150^+^ HSCs populations. Solid lines represent the linear fit of data. Dotted lines represent 95% confidence intervals. Data are presented as average ± SD, a summary of three independent experiments, *n* ≥ 3 mice per group; *p < 0.05, **p < 0.01, ***p < 0.001

Next, following the robust response to LPS (Fig. 1), we examined the dose-dependent effect of LPS on the expression of CD69. We injected mice with various doses of LPS (0.0001–20 µg) and analyzed the expression of CD69 in different hematopoietic cell populations in the BM after 2 h. CD69 expression in the LSK and the LSKCD48^−^CD150^+^ HSCs populations was significantly upregulated at higher doses of LPS (0.1 µg onwards), with maximal upregulation observed at 1 µg of LPS at 2 h. More specifically, in the LSK population, 50% of cells expressed CD69 at 0.1 µg of LPS, with expression levels reaching > 80% with 1 µg of LPS (Fig. 3A, B). Similarly, CD69 expression in the LSKCD48^−^CD150^+^ HSCs population increased from 25 to 60% with similar LPS doses (0.1 µg and 1 µg, respectively, (Fig. 3A–C). We found EC50 value of 0.07 µg (LSK) and 0.21 µg (LSKCD48^−^ CD150^+^ HSCs), and identified a low-threshold of 0.01 µg LPS for CD69 elevation in these populations. Nevertheless, we gained a clear dose-dependent response of CD69 expression at higher LPS concentrations that fit well with a linear regression (Fig. 3D, E; Additional file 1: Fig. S3 F). The threshold for LPS was possibly higher in the Lin^−^ and LK populations, with the overall increment reaching up to 20% with 20 µg of LPS (Additional file 1: Fig. S3A–C, F). Despite having a lower maximal activation in the Lin^−^ or LK, the EC50 values of 0.39 µg (Lin^−^), and 0.17 µg (LK) are in the same range as LSK or HSCs (Additional file 1: Fig. S3D–E). Taken together, HSCs and progenitors reveal similar sensitivity and very fast response to LPS as measured by the elevated surface levels of CD69.

### CD69 and CD317 expression in activated stem and progenitor populations positively correlates with the exit from quiescence

**Fig. 4:**
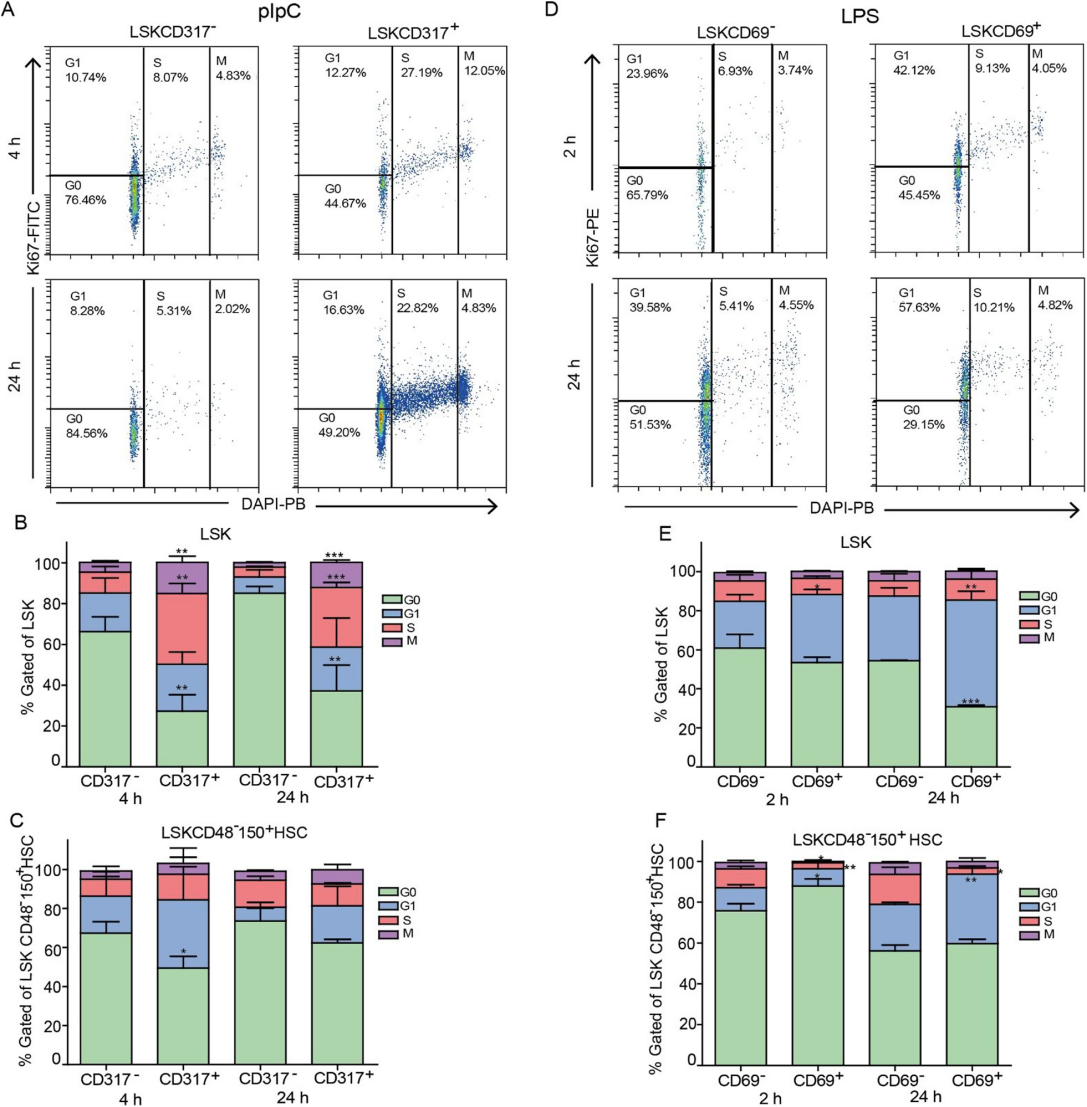
CD69 and CD317 expression on activated stem and progenitor populations positively correlate with exit from quiescence. **A** Representative FACS plots showing cell cycle analysis using DAPI and intracellular expression of Ki67 in LSK CD317^−^ (left panel) and LSK CD317^+^ (right panel) cells from pIpC-stimulated mice at 4 and 24 h after injection. **B**, **C** Quantification of cell cycle phases (G0, G1, S, and M) in CD317^−^ and CD317^+^ fractions of the LSK and LSKCD48^−^150^+^ HSCs populations from pIpC-stimulated mice at 4 h and 24 h post-injection. **D** Representative FACS plots showing cell cycle analysis using DAPI and intracellular expression of Ki67 in LSK CD69^−^ (left panel) and LSK CD69^+^ (right panel) cells from LPS-stimulated mice at 2 and 24 h after injection. **E**, **F** Quantification of cell cycle phases (G0, G1, S, and M) in CD69^−^ and CD69^+^ fractions of the LSK and LSKCD48^−^ CD150^+^ HSCs populations from LPS-stimulated mice at 2 h and 24 h post-injection. Data are presented as average ± SD, a summary of three independent experiments, *n* ≥ 3 mice per group; *p < 0.05, **p < 0.01, ***p < 0.001

We conducted a cell cycle analysis to evaluate whether elevation in activation markers may correlate with hematopoietic stem and progenitor cells exit from quiescence. In this set of experiments, we tested for Ki67 expression in pIpC- and LPS-stimulated mice at 4 and 24, or 2 and 24 h after respectively (Fig. 4A–F). Importantly, the complete cell division takes longer, thus we focused on the earlier marker of cell-cycle state. At designated time points, we extracted the BM, stained for surface markers, fixed and permeabilized the cells, and then stained for the nuclear Ki67 and DNA- content (see methods). Surprisingly, at 4 h post pIpC stimulation, a significant proportion of LSK CD317^+^ cells, but not LSK CD317^−^ cells, were in the S and M phase of the cell cycle (Fig. 4B). The CD317^+^ fractions of LSK and HSCs populations had fewer cells in the G0 phase and this trend was sustained at 24 h (Fig. 4B, C). Similar differences between cell cycle stages of CD317^+^ and CD317^−^ cells were also observed for Lin^−^ and LK populations (Additional file 1: Fig. S4 B, C). We also tested the correlation between CD69 expression and Ki67 following stimulation with LPS. Here, only a slight change was observed in the LSK population at 2 h, reaching significance at 24 h, with CD69^+^ being less quiescent (Fig. 4E). Surprisingly, we observed more G0 cells in the CD69^+^ fraction than in their CD69^−^ counterparts within the LSKCD48^−^CD150^+^ HSCs compartment 2 h after stimulation (Fig. 4F). At 24 h, the CD69^+^ HSCs were not significantly different than the CD69^−^ HSCs, and their respective G0 fractions were almost equal (Fig. 4F). As for the Lin^−^ and LK populations, their CD69^+^ fractions exhibited more Ki67^+^ than CD69^−^ 2 h after the stimulation, but these differences were largely diminished at 24 h (Additional file 1: Fig. S4E, F). Taken together, with one peculiar exception of HSCs at the early 2 h time point, our data demonstrate a positive correlation between the expression of surface activation markers and the exit from quiescence of stem or progenitor cells following pIpC or LPS stimulation.

## Discussion

This study presents the rapid activation of HSCs following immune activation in vivo. Utilizing surface markers, we demonstrate that HSCs gain rapid activation as early as 2 h after stimulation. We demonstrate the high sensitivity of primitive hematopoietic cells to pIpC and LPS, with a dose-dependent response range and a low threshold. Finally, we indicate a correlation between the expression of the activation markers and cell cycle progression in these cells, suggesting fast exit from quiescence.

Immune responses can be rapid, showing pronounced elevation of systemic inflammatory cytokines even before 2 h [30, 31]. HSCs may sense immune activation both directly and indirectly, involving multiple receptors for microbial products (TLRs) and cytokine receptors for IL-1β, IL-6, TNF-α, and IFN-γ [11, 32]. As the crucial role of HSCs is to produce more hematopoietic cells, previous studies focused on their proliferation and differentiation following immune stimulation [11]. Surface activation markers allow us to quantify earlier activation stages with a single-cell resolution (Fig. 1). CD317 (BST2) has a role in anti-viral response [33], and it has also been shown to play a role in the retention of HSCs within the BM [29]. CD69 is a major activation marker and a metabolic regulator of immune cells [34]; intriguingly, CD69 had also been implicated in HSCs and Progenitors (HSPC) retention in the BM [35], suggesting that CD69, like CD317, may regulate BM localization of these cells following stimulation. Our data is showing directly that CD69 upregulation precedes at 2 and 4 h, while CD317 upregulation is a bit slower yet remains high when CD69 decreases at 24 h. This may suggest a dynamic process of fine niche re-localization, in agreement with Florez et al. [29].

Realizing the fast activation of HSCs allows us to position them at the front line of the immune response, even within their BM niche. HSCs have been previously suggested to have a front-line role in peripheral secondary lymphoid tissues [36]. We focused on the BM, where most adult HSCs reside. Intriguingly, the sensitivity of HSCs is comparable to that of the hematopoietic progenitors in the BM (Additional file 1: Fig. S2 and 3), and to other effector immune cells in vivo [37–39], suggesting a coordinated systemic response. Our data suggest that HSCs are an integral part of the immune system. Despite recent publications suggesting no contribution of HSCs to emergency hematopoiesis [9, 10], the activation markers demonstrate these HSCs are not just a reservoir for the late stages, but instead a proximal part of an acute immune response.

## Conclusions

The activation of HSCs was suggested to affect potency and skew differentiation-bias. Trained immunity, the adapted immunity of myeloid cells, suggests fine-tuning of the immune response [25]. It will be interesting to realize how deep trained immunity stretches within the hematopoietic hierarchy. Our data suggest that HSCs do sense and gain rapid activation at a low threshold, providing possible "training" through multiple immunological stimulations throughout life. Intriguingly, HSCs' aging is intrinsic [40], but not yet fully characterized at the molecular level. HSCs were previously suggested to count cell divisions [41, 42], and their proliferation must be tightly regulated. Indeed, we recently reported that HSCs might ignore an acute hypersensitivity immune response [43]. Importantly, blocking inflammation was shown to attenuate its deleterious impact on HSPCs [44], providing strong support for prophylactic treatments. This study quantifies the rapid response of HSCs. It offers further options for prospectively studying and modulating the activation of our long-term multipotent reservoir for blood and immune cells.

## Supplementary Information

**Additional file 1.** Supplementary figures.

## Data Availability

All data generated or analyzed during this study are included in this published article and its supplementary information files.

## Abbreviations

HSCs: Hematopoietic stem cells
HSPC: HSCs and progenitors
BM: Bone marrow
pIpC: Poly-Inosinic-poly-Cytidylic
LPS: Lipopolysaccharide
SPF: Specific pathogen-free
I.P.: Intraperitoneally
LSK: Lineage-Sca1+cKit+
LK: Lineage-Sca1-cKit+
