# Supplementary material for: Rapid activation of hematopoietic stem cells
Source: Stem Cell Res Ther. 2023 Jun 6;14:152. doi: 10.1186/s13287-023-03377-6 (PMC10245525; doi:10.1186/s13287-023-03377-6)
Supplement: Supplementary file 1 — Additional file 1. Supplementary figures. [file 13287_2023_3377_MOESM1_ESM.pdf]

Supplementary figure legends:

Fig.S1 Early activation of hematopoietic progenitor cells upon immune stimulation. (A) Representative FACS plots showing the percentage of $CD69^+$ and $CD317^+$ in the $Lineage^-$ (Lin^-, left panels) and $Lineage^-$ $cKit^+$ (LK, right panels) populations from PBS-treated (control) and pIpC- stimulated mice at different time points. (B, C) Quantification of CD69 and CD317 surface expression in Lin^- and LK populations from the BM of pIpC-treated mice at different time points. (D) Representative FACS plots showing the percentage of $CD69^+$ and $CD317^+$ in the $Lineage^-$ (Lin^-, left panels) and $Lineage^-$ $cKit^+$ (LK, right panels) populations from PBS-treated (control) and LPS- stimulated mice at different time points. (E, F) Quantification of CD69 and CD317 surface expression in Lin^- and LK populations from the BM of LPS-treated mice at different time points. Data are presented as average ± SD, a summary of three independent experiments, n≥3 mice per group; * - $p<0.05$, ** - $p<0.01$, *** - $p<0.001$.

Fig.S2 Dose-response of pIpC on activation of hematopoietic progenitor cells. (A, C) Quantification of $CD317^+$ fractions in Lin^- and LK populations in the BM of mice treated with PBS (control) or various doses of pIpC. (D, E) Linear regression model fitting the dose-dependent response with the expression of CD317 in Lin^- and LK populations. Solid lines represent the linear fit of data. Dotted lines represent 95% confidence intervals. (F) Mean Fluorescence Intensity of indicated populations in response to indicated doses. Data are presented as average ± SD, a summary of three independent experiments, n≥3 mice per group; * - $p<0.05$, ** - $p<0.01$, *** - $p<0.001$.

Fig.S3 Dose-dependent activation of hematopoietic progenitor cells by LPS. (A) Representative FACS plots showing the percentage of $CD69^+$ cells in the Lin^- (upper panels) and LK (lower panels) populations from the BM of PBS-treated (control) mice and mice treated with various doses of LPS (0.01 µg -100 µg) at 2 h. (B, C) Quantification of $CD69^+$ fractions in Lin^- and LK populations in the BM of mice treated with PBS (control) or various doses of LPS. Quantification of $CD69^+$ fractions in Lin^- and LK populations in the BM of mice treated with PBS (control) or various doses of LPS. (D, E) Linear regression model fitting the dose-dependent response with the expression of CD69 in Lin^- and LK populations. Solid lines represent the linear fit of data. Dotted lines represent 95% confidence intervals. (F) Mean Fluorescence Intensity of indicated populations in response to indicated doses. Data are presented as average ± SD, a summary of three independent experiments, n≥3 mice per group; * - $p<0.05$, ** - $p<0.01$, *** - $p<0.001$.

Fig.S4 Activation of hematopoietic stem- and progenitor cells is linked with exit from quiescence. (A) Representative FACS plots showing cell cycle analysis using DAPI and intracellular expression of Ki67 in $CD317^-$ and $CD317^+$ fractions of Lin^- (upper panel) and LK (lower panel) cell populations from the BM of pIpC-stimulated mice at 4 h and 24 h post-injection. (B, C) Quantification of cell cycle phases (G0, G1, S, and M) in $CD317^-$ and $CD317^+$ fractions of the Lin^- and LK cell populations from pIpC stimulated mice at 4 h and 24 h post-injection. (D, E) Representative FACS plots showing cell cycle analysis using DAPI and intracellular expression of Ki67 in $CD317^-$ and $CD317^+$ fractions of Lin^- (upper panel) and LK (lower panel) cell populations from the BM of LPS-stimulated mice at 2 h and 24 h post-injection. (E, F) Quantification of cell cycle phases (G0, G1, S, and M) in $CD317^-$ and $CD317^+$ fractions of the Lin^- and LK cell populations from pIpC-stimulated mice at 2 h and 24 h post-injection. Data are presented as average ± SD, a summary of three independent experiments, n≥3 mice per group; * - $p<0.05$, ** - $p<0.01$, *** - $p<0.001$.

Fig.S1

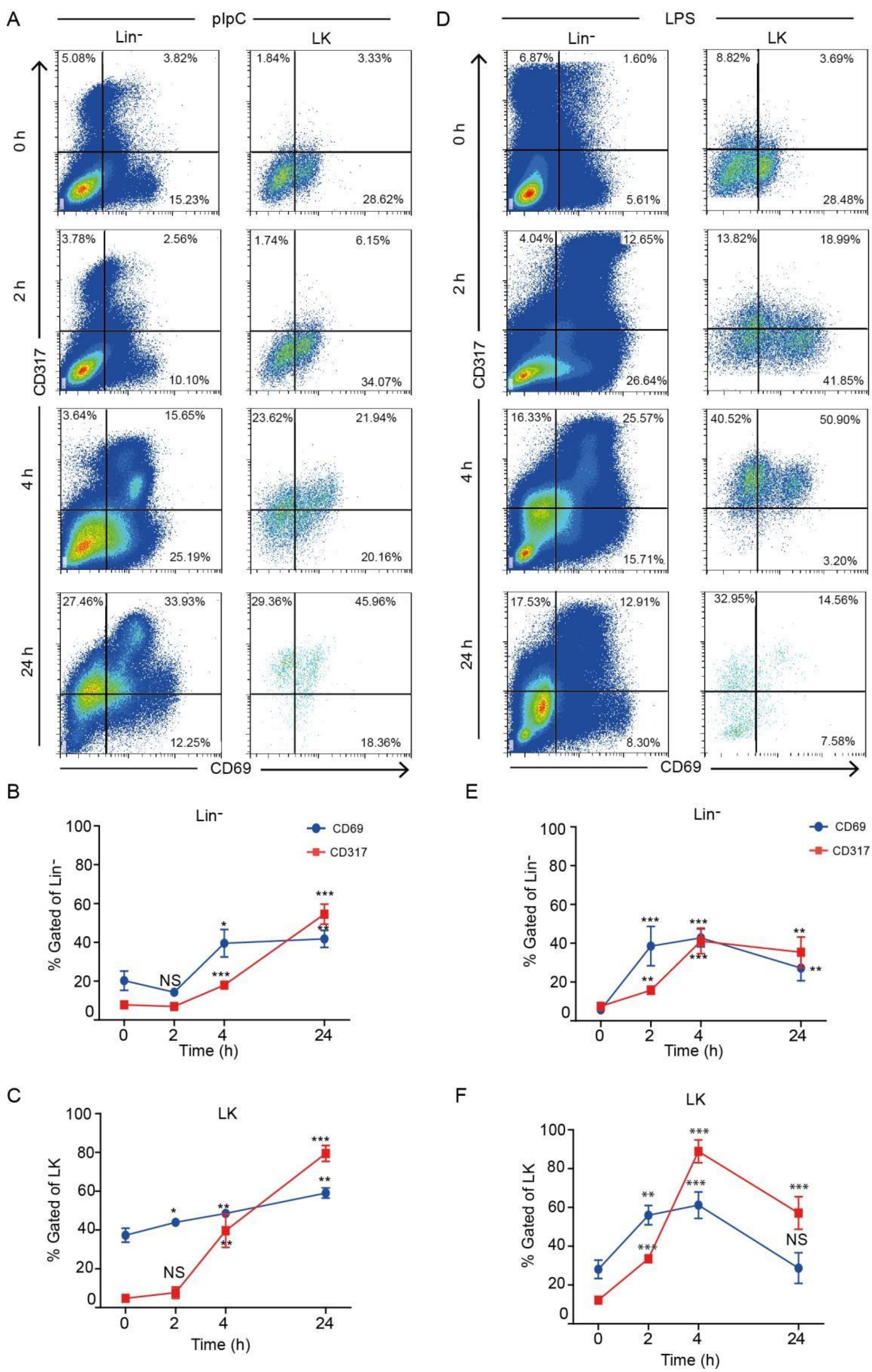

A
pIpC
Lin-
LK
0 h
2 h
4 h
24 h
CD317
CD69
5.08%
3.82%
15.23%
1.84%
3.33%
28.62%
3.78%
2.56%
10.10%
1.74%
6.15%
34.07%
3.64%
15.65%
25.19%
23.62%
21.94%
20.16%
27.46%
33.93%
12.25%
29.36%
45.96%
18.36%
D
LPS
6.87%
1.60%
5.61%
8.82%
3.69%
28.48%
4.04%
12.65%
26.64%
13.82%
18.99%
41.85%
16.33%
25.57%
15.71%
40.52%
50.90%
3.20%
17.53%
12.91%
8.30%
32.95%
14.56%
7.58%
B
E
% Gated of Lin-
Time (h)
CD69
CD317
NS
C
F
LK
% Gated of LK

Fig.S2

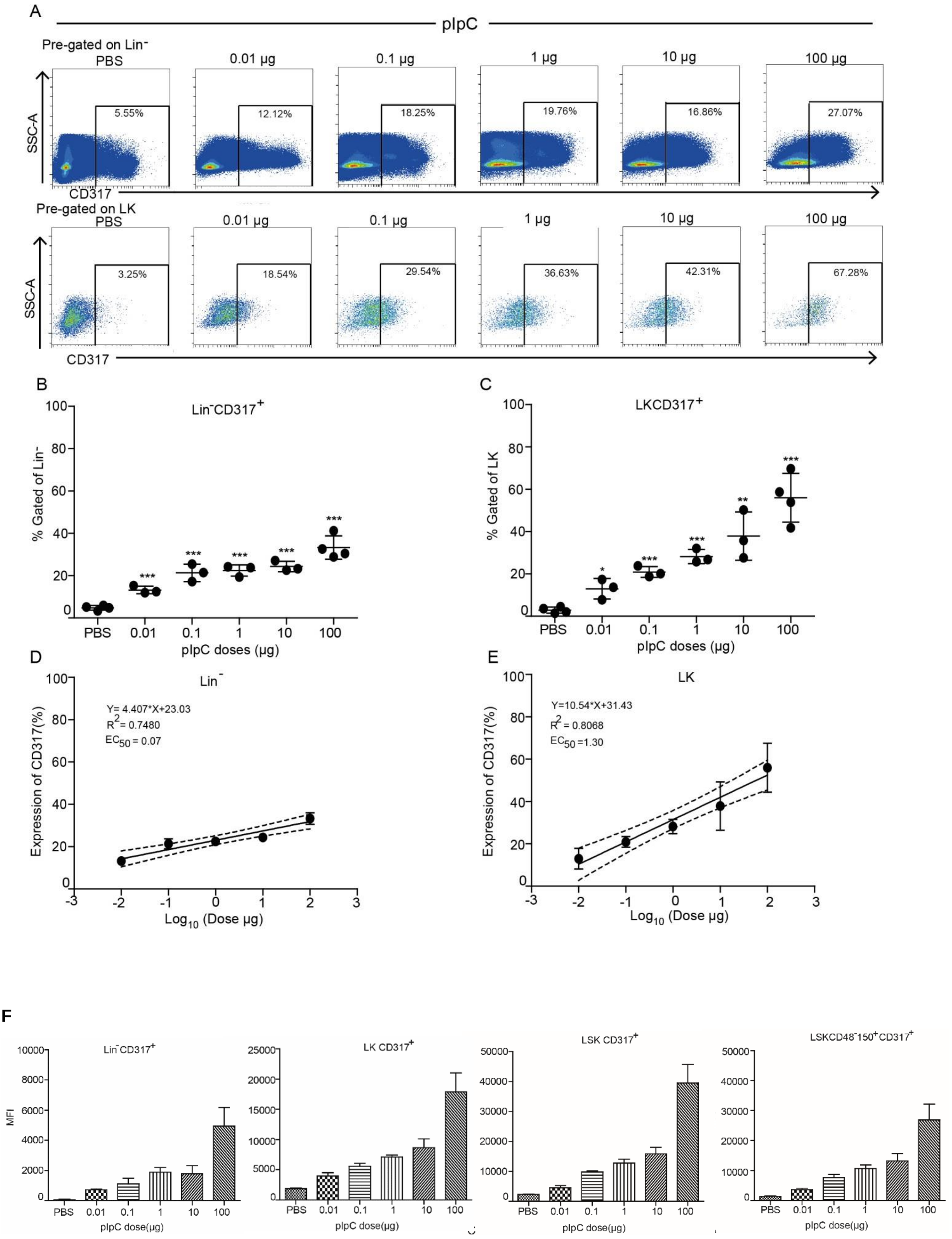
A
pIpC
Pre-gated on Lin⁻
PBS
0.01 μg
0.1 μg
1 μg
10 μg
100 μg
5.55%
12.12%
18.25%
19.76%
16.86%
27.07%
SSC-A
CD317
Pre-gated on LK
3.25%
18.54%
29.54%
36.63%
42.31%
67.28%
B
Lin⁻CD317⁺
% Gated of Lin⁻
pIpC doses (μg)
C
LKCD317⁺
% Gated of LK
D
Lin⁻
Y= 4.407*X+23.03
R2= 0.7480
EC50= 0.07
Expression of CD317(%)
Log10 (Dose μg)
E
LK
Y=10.54*X+31.43
R2= 0.8068
EC50=1.30
F
Lin⁻CD317⁺
LK CD317⁺
LSK CD317⁺
LSKCD48⁻150⁺CD317⁺
MFI
pIpC dose(μg)

Fig.S3

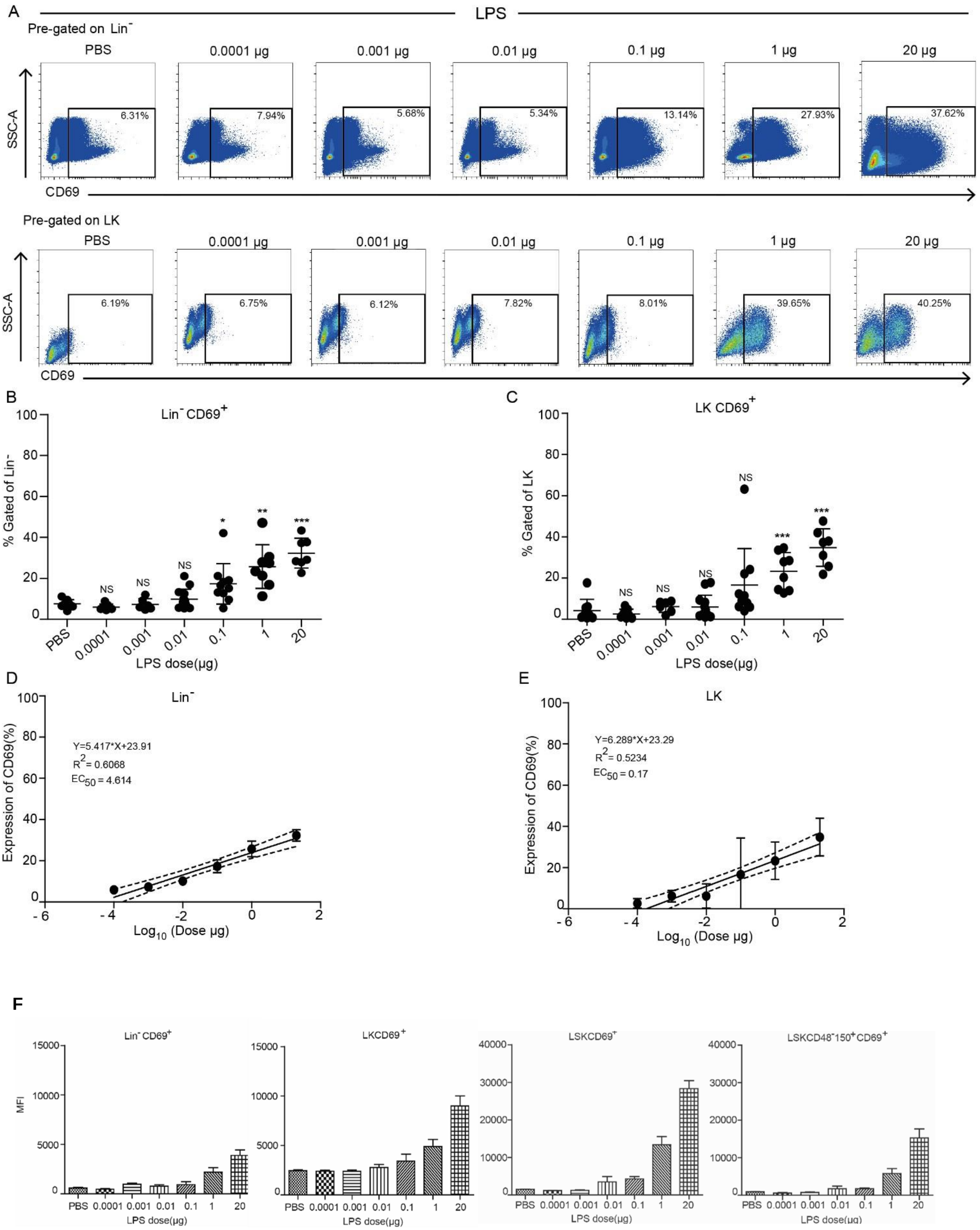

A
LPS
Pre-gated on Lin⁻
PBS
0.0001 μg
0.001 μg
0.01 μg
0.1 μg
1 μg
20 μg
SSC-A
CD69
6.31%
7.94%
5.68%
5.34%
13.14%
27.93%
37.62%
Pre-gated on LK
6.19%
6.75%
6.12%
7.82%
8.01%
39.65%
40.25%
B
Lin⁻ CD69⁺
% Gated of Lin-
LPS dose(μg)
C
LK CD69⁺
% Gated of LK
D
Lin⁻
Expression of CD69(%)
Y=5.417*X+23.91
R² = 0.6068
EC50 = 4.614
Log10 (Dose μg)
E
LK
Y=6.289*X+23.29
R² = 0.5234
EC50 = 0.17
F
Lin⁻ CD69⁺
LKCD69⁺
LSKCD69⁺
LSKCD48⁻150⁺CD69⁺
MFI
LPS dose(μg)

Fig.S4

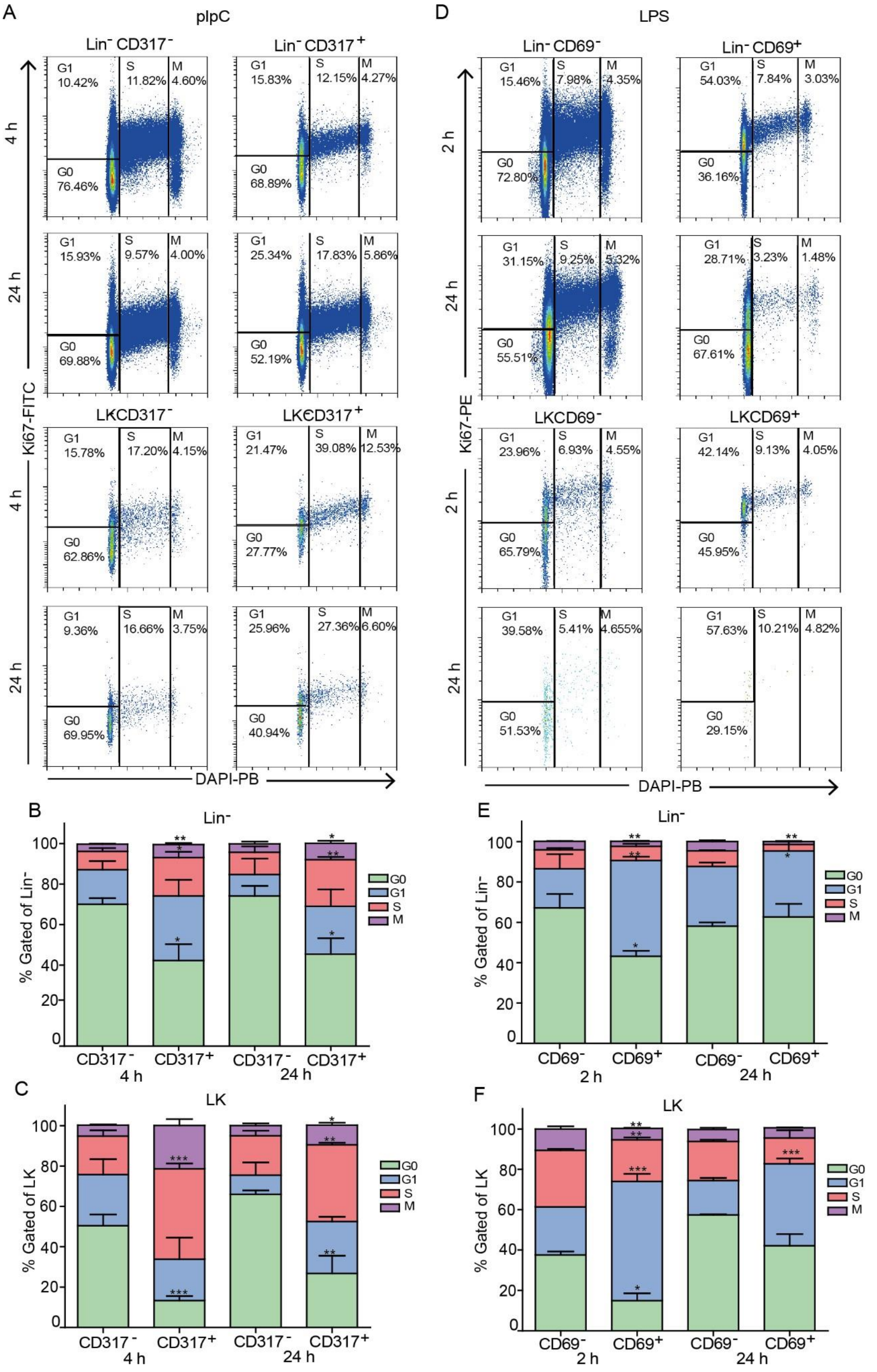

A
pIpC
Lin⁻ CD317⁻
Lin⁻ CD317⁺
LKCD317⁻
LKCD317⁺
Ki67-FITC
DAPI-PB
4 h
24 h
D
LPS
Lin⁻ CD69⁻
Lin⁻ CD69⁺
LKCD69⁻
LKCD69⁺
Ki67-PE
2 h
24 h
B
Lin⁻
% Gated of Lin⁻
C
LK
% Gated of LK
E
F
G0
G1
S
M
